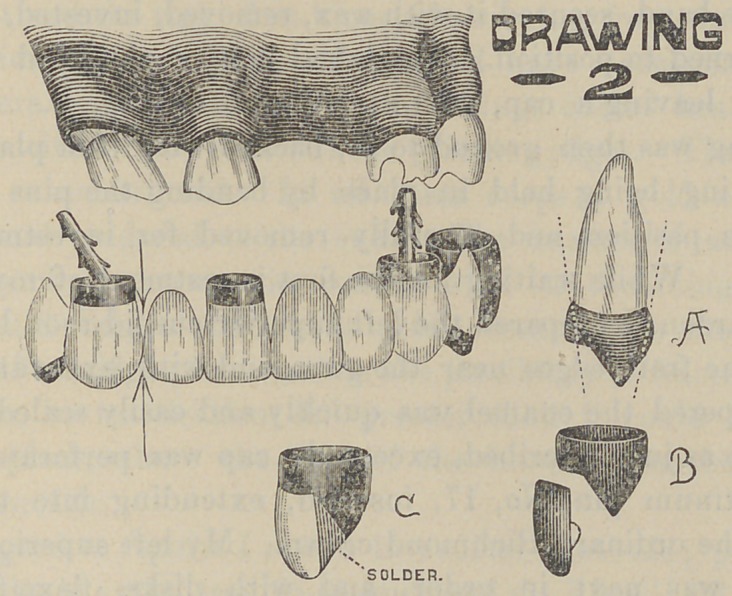# Crown and Bridge Work

**Published:** 1892-09

**Authors:** Grant Mitchell

**Affiliations:** Canton, O.


### Crown and Bridge-Work.

BY GRANT MITCHELL, D.D.S., CANTON, O.

Read before the Northern Ohio Dental Society, Cleveland, May, 1892.

I appreciate the honor conferred on me by your committee in appointing me to a place on your programme for this meeting of the Northern Ohio Dental Association.

I feel that it is one of the greatest of privileges. It affords opportunities for learning and improvement that can be had in no other way. Points that are brought out in the discussion of one's own papers are impressed more deeply, I think, than in the casual hearing or reading the dissection of some stranger's work. And yet I am not without misgivings in availing myself of the privilege and must confess a purely selfish motive in accepting it; or, if I may inflict another man's joke, my reason for consenting to appear in this role to-day, is because I feel that I can "do the greatest good to the greatest number. And that number is notoriously No. 1."

The subject of this paper, Crown and Bridge-Work, is, to my mind, second only, in importance, to that of tooth saving. Its merit is shown in the steadily increasing popularity. Invented probably prior to 1840; lost sight of for a period; and again taken up some eight or ten years ago, since when its development has been of the healthiest possible kind. Crown and bridge-work is no longer a theory, but has grown to be a *fact*, that in the hands of skillful dentists will change our system of prosthetic dentistry.

I said my subject was *second* to that of tooth saving; let me retrace, and say that it is, indeed, an aid to, and a part of that grandest work intelligent dentistry aims to accomplish. For what nearer approach to the *perfect* tooth saver have we than a well-made and properly-fitted crown—be it cap or pivot?

There is a multitude of methods for doing this class of work, and so much has been said and written on the subject that

I feel a description of any of the methods would be but a repetition, and exceedingly superfluous, except, in so far as it may be an intimation of what has seemed to me to be good.

I have, as a general thing, but little sympathy with those *curiosities* that are occasionally presented under the caption of "Novel Bridges." They involve, usually, complications in their construction that lends nothing to their permanence or usefulness and in hands unskillful render the work more difficult of introduction, to the public, for those who are earnestly striving to do good, by reason of its liability to fail.

Right here it may be pertinent to remark that *popular* dental education is a subject occasionally discussed by members of our profession.

I know of no better way to accomplish that end than by doing work that will inspire confidence in our efforts and ability. There are comparatively few people, if any, who are willing to go to a considerable "expense" in having their teeth "fixed up" when the ghost of the work they have had done points to the ultimate loss of the teeth.

We may speculate on the feasibility of introducing text books in public schools, and like methods, but the education that educates is the one which demonstrates the ability of dentistry to save teeth—the one which changes the outlays of people from "expense" to investments in that which will profit them.

For these reasons I carefully avoid "juggling" in my practice. That is to say, I try not to go to extremes in experiment. My judgment suggests methods that seem to be improvements on those I had been using, which, if proven a success, after trial, I adopt.

The principles I employ in the construction of crowns and bridges, are old and simple. I am not tied, however, to any particular method. I use whatever my judgment suggests for the case in hand. If a Logan crown seems to meet requirements (and there are many cases where it answers most admirably), I do not hesitate to use it. I can not see that a Logan is weaker by having its pin baked into it, than a crown wherein it is cemented. I do not use them, however, back of the anterior six, nor would I sacrifice a fairly strong lingual wall to adapt one. Preferring in such cases to make a crown after the style of the "Richmond" and save any portion of the natural tooth that may add strength and permanence to the work.

On molar teeth, except where the progress of decay renders it utterly impracticable, I use cap-crowns. They are the best, and no morbid esthetic considerations should deter us from using that which is most durable.

In peculiar cases, where a tooth is so badly decayed or abraded that a gold filling would be very unsightly, and where the nerve is still alive and healthy, and an ordinary crown operation, or even a filling would necessitate its devitalization, I have successfully crowned in a manner suggested by Dr. Dewey, of Cleveland, by beveling from the labial to lingual aspects, and making what is practically a cap crown, to which I fit a porcelain face, and mount in the same manner as a gold cap. In such a crown we sacrifice the minimum amount of tooth substance, display but little gold, and preserve the full vitality of the tooth. It makes, too, an excellent support for a bridge, and can readily be placed on bicuspids, if it is desirable to avoid a display of gold.

I have here a specimen bridge, illustrating some of the methods of Dr. Hacker, of Indianapolis, that will appeal to us at least in the sense of durability. The piece I will hand to you for inspection has a cuspid crown made somewhat on the principle just described, with the difference that the porcelain facings are *cemented in* instead of being soldered to it. (I would say by way of explanation, that the spaces you will notice on the palatal side of the piece, were left so purposely in order to show how it is put together. In practice these spaces would be filled flush with solder.)

The construction of this kind of a bridge is, briefly, as follows: The roots and crowns to be used as anchorages are prepared in the usual manner. Ordinary plain teeth of suitable sizes may be selected. The pins ground off as shown in the illustration (A. B. Fig. 1). Stirrups of gold are then shaped around them; using about No. 14 or 16 gauges at the cutting or

Fig. 1.

grinding edges (C. Fig. 1). and No. 24 for hoops, Trim to contour, and solder to back of plates; making shells very heavy and thick enough to do all necessary contouring. Mount on the model and solder. Fill the shells with cement, fit in the fronts and round off the edges.

We thus have a strong bridge, on which none of the porcelains have been split or checked in soldering.

My usual practice, however, in constructing a bridge piece is that which is probably used by the majority, and is, I think, on the whole, the simplest, most reliable and certainly the most artistic.

As I made a piece, recently, that called into practice nearly all of the principles connected with this style of denture, I will describe the case.

A lady called at my office with the right superior first bicuspid, right superior lateral, left superior central and lateral and left superior first bicuspid missing. The right superior cuspid through a peculiarity of the occlusion had been tilted forward in its socket, almost closing the space occasioned by the loss of the lateral incisor. The central was abraded to more than half its length, and the left superior cuspid was decayed, and broken off near the gum. The other teeth, the first molar on the left, and second bicuspid, and first molar on the right were in fair condition.

My first step was to tap the right superior cuspid and make an application to devitalize the pulp. At the same sitting I prepared the other abutments for the bridge by grinding square the abraded edges and truing the sides of the central with corundum wheels and disks. I next beveled the labial side leaving a slight shoulder near the gum, as shown in drawing 2, Fig. A.

A strip of block-tin, rolled to about No. 30 gauge, and a quarter to five-sixteenths of an inch in width, by one and a half inches in length, trimmed to approximate the festoon of the gum, was placed around the tooth as prepared, drawn close and held in position by the fingers of the left hand. With a pair of flat-nosed pliers the ends of the tin band were grasped in such a manner as to draw it to an accurate fit. The band was then withdrawn, carefully straightened, and the ends cut just outside the marks left by the nose of the pliers. I then cut a strip of gold from the pattern thus made, beveled the ends on opposite sides with a hammer and anvil, bent into the form of a hoop, till the ends overlapped, and soldered with 20k gold.

I thus made a band that *fitted* the tooth *at the neck*—the place where the band ought to fit, and where *wire* patterns without the use of remarkable judgment, cannot succeed in doing it, owing to the fact that the labial aspect of the tooth is usually much higher than the lingual, and a pattern much too large is the result. Nor do I believe—without seeing—that gentlemen who fit the gold in the mouth, using no pattern, do quite so well, because of the lesser degree of pliability in the gold.

My band was then beveled at the cervical edge and *driven* on—this, sometimes, requires no little force. I next burnished a plate of pure gold over the end and beveled surface of the tooth, inside the band, secured it with wax, removed, invested, soldered and returned to position. The labial side of the band was then trimmed, leaving a cap, such as is shown at Fig. B. A porcelain facing was then ground to fit, backed with thin platinum—the backing being held in place by bending the pins over it,* waxed in position, and carefully removed for investment and soldering. While waiting for the first investment of my central cap to harden, I prepared the left superior cuspid root by grinding off the frail edges near the gum, and with excavators suitably tempered the enamel was quickly and easily scaled off. A cap made as just described, except the cap was perforated and a pure platinum pin, No, 17, inserted, extending into the root, making the ordinary Richmond crown. My left superior second bicuspid was next in order, and with disks, flexo-files and corundum wheels of various sizes, this was soon reduced to the proper shape for cap-crowning. In all cases using the block-tin patterns.

My patient was then dismissed to await the action of the arsenical application—returning next day with the nerve slightly sensitive, yet sufficiently dulled to sensibility to admit of its extirpation.

I then excised the crown, made a Richmond crown as described, with a pin standing off at a decided angle, as shown in the illustration.

My caps and crowns were all placed in position and a plaster impression taken, from which a plaster-and-marble-dust model

* My experience has demonstrated that this is the best way to hold the platinum backing securely in position, and it does *not* cause checking or cracking of the teeth as is sometimes claimed. If the platinum is permitted to *overlap* the ends of the tooth and gold flowed there over the contraction of the gold, on cooling, will surely crack the porcelain and that is about the only way it can be cracked—ordinary care only is necessary in heating and cooling.

was made. Dummys, backed in the usual manner, by burnishing thin platinum over the lingual side, investing, and melting coin scraps over the platinum surfaces, were mounted on the model; the whole *invested* as one piece and the parts united with solder (using care, however, not to unite the right superior cuspid with the lateral incisor adjoining—making two separate pieces of it—the right lateral incisor *swinging* from the central abutment and the right first bicuspid swinging from the cuspid.)

In bicuspid and molar dummys I have occasionally seen cases where ordinary *plate* teeth, or facings *backed* heavily were used, but without protection at the grinding edges. They should never be so constructed. In all cases cusps of gold should be swaged and soldered to the ends. The surface of any tooth, front or back, that comes in contact with its occluding antagonist, should be protected with gold, because the comparative rigidity of a bridge-piece renders the possibility of fracture of the porcelains, in mastication, far greater than it could be in the case of a plate, where the force of the blow is absorbed in soft tissues beneath.

In regard to *repairs* I resort to radical methods; preferring to take a little extra trouble at once and do it well, rather than spread it out over time by trying to "fix it" *in the mouth* and having to repeat the fixing at almost regular intervals. I *take the bridge out*, repair it, and replace it. If it has been properly constructed the necessity for repairs occurs so infrequently that I can well afford to do this even at a sacrifice of some slight remuneration.

I frequently use bands for attachments. I like them better than to sacrifice a comparatively good crown. But I make them sufficiently heavy even at the expense of appearance, and recently I have been giving them additional strength by tapping and setting one or two gold *anchor screws* on the palatal side and, perhaps, one on the disto-labial. Screws in this connection open an interesting field of speculation as to the extent to which their use may be advantageously carried. Space, however, will permit only the mere suggestion of this interesting feature at this time.

There are some operators who buy "Seamless bands" and ready-made cap crowns. I want to enter a protest against the use of such articles. I have infinitely less respect for ready-made bands or gold crowns, however limitless the variety, than I have for ready-made clothes. The fit may seem good to the man whose want of experience leads him to use such things, but it is only a matter of time 'till your bands and crowns "split up the back" and "bag at the knees" in a manner most damaging. A crown that is a shade too large may be "drawn in" until it hugs the root so tight that it is difficult to withdraw it, but that is *not fitting*. It must be so accurately made from an accurate pattern that a burnisher will *stretch it* into the little depressions without kinking, and that cannot be done with a band that was "drawn in."

I know no reason why men practicing *dentistry* should buy such things. The time necessary to construct a gold cap should not exceed thirty minutes, and the average cost of a molar crown with solid gold cusps, "made to order," is about one dollar and sixty cents. If you cannot easily make your gold bands and crowns, you cannot possibly make bought ones fit.

DISCUSSION.

Dr. J. E. Robinson: After a careful reading of Dr. Mitchell's paper, a copy of which was kindly sent me, I find so few points to attack, that in a general way my "opening discussion" must partake more of the character of an indorsement than of adverse criticism. Still, with the paper before me I am enabled to find a few points that, had I written it, would have been treated a little differently, though, perhaps, not so ably. Although the Doctor further on in his paper admits that crowning teeth and portions of teeth, helps to preserve them for further usefulness and renders what would otherwise be unserviceable, capable of renewing its functions, I must insist that crown-work is in fact tooth-saving and not "second only in importance to tooth-saving." To my mind crown-work, especially cap crowns, when made to take the place of large fillings in molar and bicuspid teeth, where on account of frail walls or inability to obtain good anchorage is tooth-saving par excellence. If carefully fitted and the shell or band made to extend beyond and well under the margin of the gums, teeth when largely decayed can be so crowned that good service can be had much longer than any filling of whatever material constructed can possibly do. Hence I call this tooth-saving. In regard to using porcelain crowns of the Logan or Bonwill stamp, I, too, am of the opinion that none but the six anterior teeth are so well or nearly saved, as by the shell or cap crown. Even in these teeth much better results are obtained if the tooth is supported by a small band around the neck as well as by the post in the center of the tooth. The band on this class of teeth can be fitted so nicely and trimmed so closely that when finished it will have the appearance of cervical filling and be no more conspicuous.

I have never crowned a bicuspid or seen one done by other operators that I did not think would have been better with the addition of a band. The different methods of setting crowns are in the main orthodox, and I will take none of your time in what must be a repetition of manipulation.

The beveling of the tooth where the nerve can be saved by so doing is good practice, as we are enabled to do away with a portion of the gold face which is more or less unsightly, and supply its place with porcelain which, when carefully selected, can be made to harmonize with the surroundings.

On bridges all facings should be protected, and that can be accomplished without bringing the gold into prominence by a slight bevel at the cutting edge. Removable bridges are to me an abomination, so I will pass that portion of the paper with no further comment.

As a rule 'tis better to remove a bridge when a tooth or facing becomes broken before attempting to repair it, but often we are compelled to try to replace a facing with the bridge in place. I have here a few instruments suggested by myself and made by Dr. J. F. Stephan that have been of great help in such cases. You will see that by their aid after you have fitted the facings and drilled holes for the pins, that they can be made quite secure, in fact quite as secure as if riveted, which is the result practically attained. Perhaps it would be superfluous for me to add that whenever possible all bands should project to some extent over the cutting edge of the tooth to prevent pushing up through force of mastication. With these few remarks I leave the paper to be discussed in its other and perhaps more important parts to those who follow me.

Dr. J. F. Dougherty agreed with Dr. Mitchell in his methods of crown and bridge-work, but did not approve of bands generally in bridge-work.

Dr. G. Mitchell does not use bands back of the anterior teeth, but thinks they will not creep up under the gum and cause irritation if they are properly fitted to the crown of the tooth.

Dr. G. H. Wilson said if a shoulder be ground into the lingual surface of a cuspid and the band fitted approximately, with the aid of the cement, it would not be so liable to displacement.

Dr. J. R. Owens thought that crown and bridge-work was being overdone. Of nine cases in ten where it is used it should not be. To band two teeth to replace one was not wise as it was only a matter of time until the banded teeth would be destroyed by decay under the bands. Gold crowns are excellent substitutes where the natural crown is gone, but to place them on teeth that could be filled, in his judgment was bad practice.

Dr. J. H. Wible thought gold crowns preferable to fillings where it was necessary to largely restore the crown.

Dr. Douds asked Dr. Mitchell how he removed a bridge when repairs were necessary.

Dr. Mitchell said if it was a band to be removed he generally used a bayonet forcep, placing one of the beaks on the edge of the tooth, the other under the further edge of the band and by gently bringing the forcep together the attachment was broken. In case of a gold cap he takes an engine bur, No. 00, and cuts a slit in the cap, folds back the corners and it is easily displaced. To reunite, bend back the corners, lay a small piece of platinum on the under side and flow solder into the space made by the bur. In case there was a post in the root he cuts off the post and removes from root by drilling around it with a suitable bur.

Dr. J. R. Bell had seen enough bridge-work to convince him that it was the most dangerous work that could be placed in the oral cavity. It not only causes local trouble but often systemic. Some of the frequent results were abscess, neuralgia, etc.

Dr. W. H. Fowler said he had returned to the old-fashioned method of clasping. Wide clasps and rubber vulcanized in the tooth to rest against ridge and a small portion of palatine side of gums.

Dr. L. L. Barber thought if properly used bridge-work was a good thing, but the operator must use much judgment as to the advisability of a bridge and also in its construction. Much of the faulty work comes through lack of judgment on the part of the operator.

Dr. H. E. Dunn said his experience with clasp plates had been a sorry one. There must be more or less movement of the plate and that only aggravated the irritation. If confined to small pieces he thought the bridge-work in suitable cases was better than filling. A nicely adapted band well burnished to the tooth will approximate a good gold filling.

## Figures and Tables

**Fig. 1. f1:**
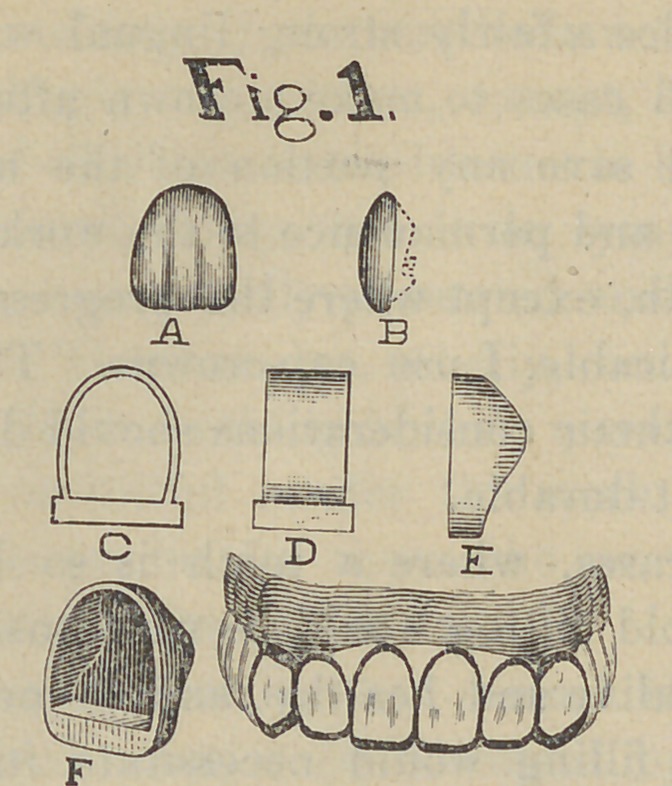


**Drawing 2. f2:**